# Effects of Slow-Release Fertilizer on Lotus Rhizome Yield and Starch Quality under Different Fertilization Periods

**DOI:** 10.3390/plants12061311

**Published:** 2023-03-14

**Authors:** Yamei Zhu, Kangming Deng, Peng Wu, Kai Feng, Shuping Zhao, Liangjun Li

**Affiliations:** 1 College of Horticulture and Landscape Architecture, Yangzhou University, Yangzhou 225009, China; zhuyamei001@163.com (Y.Z.); h844198419@163.com (K.D.); wupeng@yzu.edu.cn (P.W.); fengkai@yzu.edu.cn (K.F.); 2 Joint International Research Laboratory of Agriculture and Agri-Product Safety of Ministry of Education of China, Yangzhou University, Yangzhou 225009, China

**Keywords:** *Nelumbo nucifera* Gaertn., slow-release fertilizer, lotus rhizome yield, starch, enzyme activity

## Abstract

Slow-release fertilizer is an environmentally friendly fertilizer that is widely used in crop cultivation instead of traditional nitrogen fertilizer. However, the optimal application time of slow-release fertilizer and its effect on starch accumulation and rhizome quality of lotus remains unclear. In this study, two slow-release fertilizer applications (sulfur-coated compound fertilizer, SCU, and resin-coated urea, RCU) were fertilized under three fertilization periods (the erect leaf stage, SCU1 and RCU1; the erect leaf completely covering the water stage, SCU2 and RCU2; and the swelling stage of lotus rhizomes, SCU3 and RCU3) to study the effects of different application periods. Compared with CK (0 kg∙ha^−1^ nitrogen fertilizer), leaf relative chlorophyll content (SPAD) and net photosynthetic rate (Pn) remained at higher levels under SCU1 and RCU1. Further studies showed that SCU1 and RCU1 increased yield, amylose content, amylopectin and total starch, and the number of starch particles in lotus, and also significantly reduced peak viscosity, final viscosity and setback viscosity of lotus rhizome starch. To account for these changes, we measured the activity of key enzymes in starch synthesis and the relative expression levels of related genes. Through analysis, we found that these parameters increased significantly under SCU and RCU treatment, especially under SCU1 and RCU1 treatment. The results of this study showed that the one-time application at the erect leaf stage (SCU1 and RCU1) could improve the physicochemical properties of starch by regulating the key enzymes and related genes of starch synthesis, thus improving the nutritional quality of lotus rhizome. These results provide a technical choice for the one-time application of slow-release fertilizer in lotus rhizome production and cultivation.

## 1. Introduction

Agriculture is an important industry in the national economy, and lotus has been dominant in the production and consumption of aquatic vegetables in China [1]. By 2021, the planting area of lotus will be more than 600,000 hectares in China. Around the world, fertilizers play an important role in increasing crop yields and improving crop quality, particularly in fertilizer application rates and application stages [2,3,4]. In the production and cultivation of lotus, there are a variety of conventional fertilization methods, mainly based on topdressing, base fertilizer as a supplement. To achieve high yield, repeatedly topdressing is needed in the growth period of lotus, which is high in fertilizer consumption, time and labor [5]. This pattern of fertilization can lead to increased production costs and nutrient loss, which can lead to environmental problems such as eutrophication of water bodies [6]. Therefore, it is urgent to take measures to solve the problems encountered by this mode of production.

In the past decade, slow-release fertilizer (SRF), as an environmentally friendly fertilizer, has been widely used in crop production; because it can improve the nutrient absorption efficiency of crops, the one-time application of slow-release fertilizer can also reduce the burden of fertilizer on the environment and reduce production and labor costs [7,8]. The main slow-release fertilizers widely used in crop production and cultivation are resin-coated urea (RCU) and sulfur-coated urea (SCU). RCU mainly uses polymer resin to form a dense film outside the fertilizer so that the fertilizer is slowly released. It is an essential fertilizer for improving the light and simplified cultivation technology of crops [9,10]. SCU combines the functions of slow-release nitrogen fertilizer and sulfur fertilizer. It is made by coating urea with sulfur and polymeric microcrystalline wax sealant. Nutrients are slowly released through micro pores in the coating material to provide continuous nutrients for crops [11,12]. Therefore, the application of slow-release fertilizer in lotus production and planting process is an important measure for solving the problems of production cost, labor waste and environmental pollution.

There have been many studies on the effects of nitrogen fertilizer on crop yield and starch quality. In rice, nitrogen application can improve rice yield by increasing SPAD and net photosynthetic rate of rice leaves, increasing panicle number, grain number and grain weight [13,14], and improving rice taste quality by increasing protein content, final viscosity and setback viscosity, and reducing peak viscosity and breakdown viscosity [15,16]. In maize, application of nitrogen at different stages could significantly increase protein content and yield in corn grain [17,18], while delayed application of nitrogen significantly has increased starch and protein content, improved corn grain quality, starch grain size and starch quality [19,20]. In wheat, an appropriate amount of nitrogen fertilizer could significantly increase the number of ears and grains and increase wheat yield [21,22]. After an increase of nitrogen application rates, starch granule size, pasting viscosity and setback viscosity of wheat grain decreased significantly, while protein and small starch granule increased significantly [23,24]. Delayed application of nitrogen fertilizer could increase protein content, grain hardness and bakery quality of wheat bread [25]. However, the effect of slow-release fertilizer on crop yield and starch quality is still less studied. In wheat, a one-time application of slow-release fertilizer could significantly improve the SPAD value of wheat leaves, improve photosynthetic efficiency, increase the number of ears and grains, and thus increase wheat yield [26]. In maize, slow-release fertilizer could significantly increase SPAD, grain nitrogen content and maize yield [27]. After delayed treatment of slow-release fertilizer (six-leaf stage treatment), the starch content and the number of large starch granules of waxy maize were significantly increased and the starch pasting properties was significantly improved [28]. These results indicate that changing fertilizer application times is a strategy for pursuing high-yield, high-quality crops.

Starch is the main component of lotus rhizome, accounting for about 70% of dry weight [29]. There are two types of lotus starch granules. One is long oval starch, whose main component is amylose, and the other is nearly round starch, whose main component is amylopectin [30,31]. In addition, granule bound starch synthase (GBSS) is the key enzyme for controlling the synthesis of amylose [32,33]. Starch branching enzyme (SBE) and soluble starch synthase (SSS) are mainly related to the synthesis of amylopectin, and they ultimately affect the physicochemical properties of starch [34,35]. In rice studies, the combined application of organic and inorganic nitrogen significantly increased the activity of GBSS and SBE and their related gene expression and promoted the synthesis and accumulation of amylose and amylopectin [36]. The contents of amylose and amylopectin in rice were significantly increased under SCU treatment, the peak starch viscosity and hot pulp viscosity of rice were increased, and rice nutritional quality was improved [37]. Under urea treatment, the relative expression levels of 20 days after anthesis *TaSBE* and *TaSSS* in wheat were significantly increased, the proportion of B-type small starch granules in endosperm was increased, the relative expression levels of *TaGBSSI* and *TaGBSSII* were decreased, the biosynthesis of starch and amylose was reduced and the physicochemical properties of wheat starch were significantly affected [38]. The results indicated that the phenological stage could significantly affect starch synthesis and crop starch physicochemical properties after fertilizer treatment.

Previous studies have focused only on the effects of slow-release fertilizer on crop agronomic traits, yield and yield components. However, the effects on the quality of lotus rhizome starch remain unclear. In addition, it is still unknown whether the application time of slow-release fertilizer will affect the yield and starch quality of lotus rhizome. This study investigated the effect of two type slow-release fertilizer application times on the yield and starch quality of lotus rhizome under the same fertilization level. It explored the choice for the one-time application of slow-release fertilizer in lotus production and planting.

## 2. Results

### 2.1. Release Characteristics of SCU and RCU

**Figure 1:**
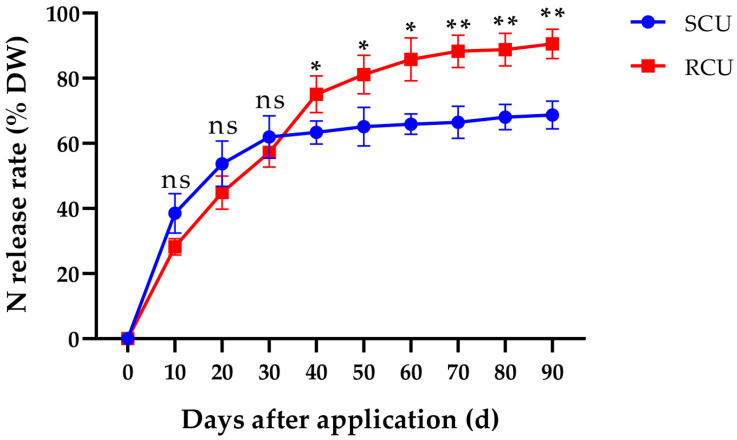
Nitrogen release rates of SCU and RCU in the pool. (ns) means not significant. (*) represents *p* values of < 0.05 and is considered statistically significant. (**) represents *p* values of < 0.01 and is considered highly statistically significant. Error bars show the standard deviation (SD) from three biological replicates.

Significant differences were observed in the N release characteristics of SCU and RCU in the pool (Figure 1). The N release rate of SCU and RCU was not significant by 0–30 days after application, showing that the N release rate was the same between SCU and RCU in the early stage. By 40, 50 and 60 days after application, the N release rates of RCU were 18.54%, 24.57% and 30.20% higher, respectively, than SCU. The nutrient release rates of the RCU by 70, 80 and 90 days after application were 32.80%, 30.41% and 31.83% higher, respectively, than SCU. RCU showed a sustained release at the middle and later stages, which was when the peak of N release appeared throughout 70–90 days after application. SCU showed a trend of sustained release at the early stage and middle stages.

### 2.2. Effects of SCU and RCU on SPAD Value and Pn

**Figure 2:**
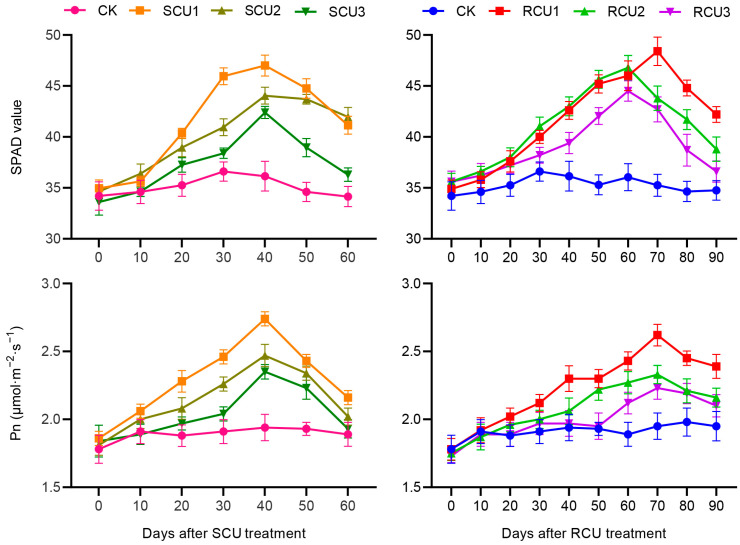
The SPAD value and Pn under SCU and RCU treatment. Error bars show the standard deviation (SD) from fifteen biological replicates.

Dynamic changes in SPAD and Pn of lotus leaves after fertilization were determined. Over time, SPAD value and Pn of lotus leaves first increased and then decreased under SCU and RCU treatments (Figure 2). After SCU and RCU application, SPAD and Pn of lotus leaves decreased gradually with the delay of fertilization stage. After SCU1 treatment, SPAD values and Pn reached maximum value (47.0 and 2.74 μmol m^−2^ s^−1^) at 40 days, which were significantly higher than CK, SCU2 and SCU3 (Table 1). Under RCU1 treatment, the maximum SPAD value (48.4) was 10.50% and 13.35% higher than RCU2 and RCU3, respectively, at 70 days. Pn reached a maximum value (2.62 μmol m^−2^ s^−1^) that was significantly higher than CK, RCU2 and RCU3 at 70 days of RCU1 treatment. The results showed that SCU and RCU application in the erect leaf stage (SCU1 and RCU1) could significantly improve the photosynthetic capacity of lotus plants.

### 2.3. Effects of SCU and RCU on Starch Content and Yield

**Figure 3:**
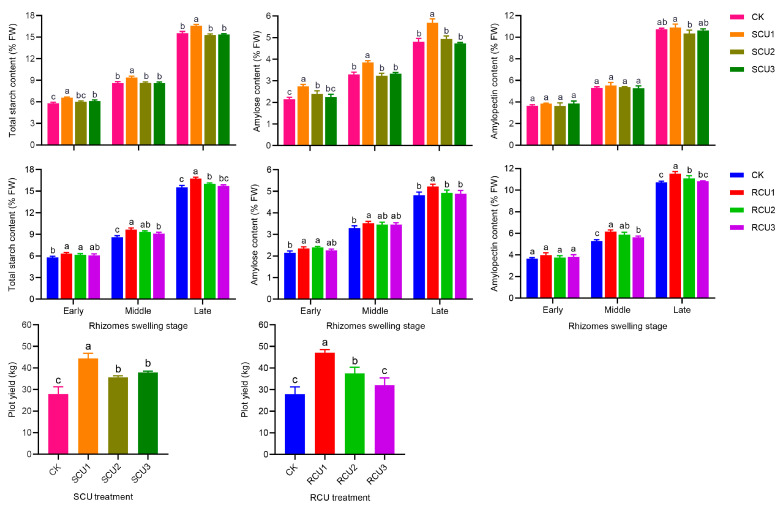
Starch content and yield under SCU and RCU treatment. Different letters indicate a significant difference in starch content and yield at the swelling stage of the lotus rhizome between different treatments at the 5% level. Error bars show standard deviation (SD) from three biological replicates.

To explain the effect of increased photosynthetic capacity on starch accumulation and starch content in lotus, we measured starch accumulation and starch content at three expansion stages. Starch content gradually decreased with the postponement of fertilization stage (Figure 3). In this study, after SCU1 treatment, total starch, amylose and amylopectin content at the later stage of lotus expansion reached its maximum value (16.58%, 5.68% and 10.90%, respectively) and total starch and amylose were significantly higher than those of other treatments, while the content of amylopectin was not significantly different than SCU2 and SCU3 treatments. Compared to CK, RCU1 substantially increased total starch, amylose and amylopectin content, reaching its maximum value (16.75%, 5.22% and 11.53%, respectively) at the late swelling stage. The results showed that starch accumulation and content decreased gradually with the delay of SCU and RCU applications. In addition, total starch content was not significant different at the rhizome swelling stage of lotus after SCU1 and RCU1 treatments, but the amylose content was significantly higher than RCU1 after SCU1 treatment and the amylopectin content was significantly lower than RCU1 after SCU1 treatment (Table 2).

Lotus rhizome yields from SCU treatments showed a trend of SCU1 > SCU3 = SCU2 > CK, while SCU1 (44.35 kg) was 16.94%, 24.49% and 58.85% higher than SCU3, SCU2 and CK, respectively. After treatment with RCU1, lotus rhizome yields reached a peak value (47.06 kg), which was 25.39%, 46.97% and 68.55% higher than RCU2, RCU3 and CK, respectively. The results showed that both SCU1 and RCU1 treatments could significantly increase lotus rhizome yield. Compared to SCU1 treatment, lotus rhizomes yield under RCU1 treatment was significantly higher than under SCU1 treatment (Table 2).

### 2.4. Effects of SCU and RCU on Starch Granule Morphology

**Figure 4:**
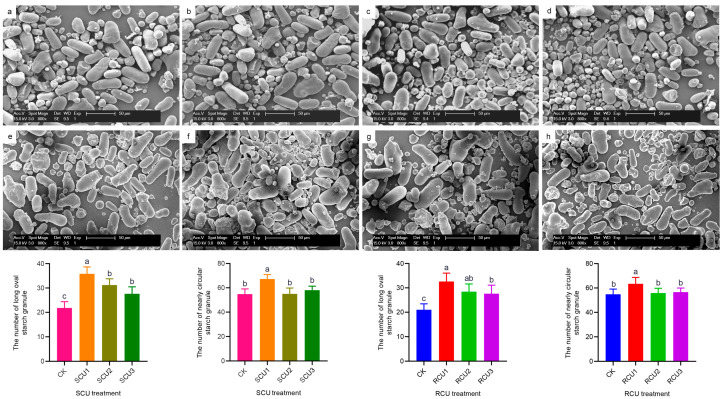
Effects of SCU and RCU on the morphology and the number of rhizome starch granules in the late swelling stage of lotus. (**a**–**d**) The granule morphology of starch under CK, SCU1, SCU2 and SCU3 treatments. (**e**–**h**) The granule morphology of starch under CK, RCU1, RCU2 and RCU3 treatments. Different letters indicate a significant difference in starch granule at the late swelling stage of the lotus rhizome between different treatments at the 5% level. We analyzed the photos to count the long oval and round granules. We analyzed the data using IBM SPSS 26.0 software for each sample of ten biological replications. Error bars show standard deviation (SD) from ten biological replicates.

In order to explore the change in starch content, we selected SCU and RCU treatments of starch granules in the late swelling stage for morphological observation and we examined the number of starch granules by using statistics (Figure 4). SCU and RCU treatments had no significant effect on the morphology and size of starch particles, but they had a significant effect on the number of the two types of starch particles (Figure 3a–h). The results showed that the numbers of long oval starch and nearly circular starch granules under SCU1 treatment reached peak value, which when ordered from largest to smallest number was SCU1 > SCU2 = SCU3 > CK and SCU1 > SCU3 = SCU2 > CK, respectively. The number of long oval starch granules treated with RCU showed a trend of RCU1, RCU3 = RCU2 and RCU1 > RCU3 > CK, and the number of nearly circular starch granules treated with RCU showed a trend of RCU1 > RCU3 = RCU2 > CK, respectively, where they reached maximum value under RCU1 treatment. These results indicated that the trend in starch granule quantity under SCU and RCU treatment was consistent with starch content at the late swelling stage of the lotus rhizome.

### 2.5. Effects of SCU and RCU on Starch Pasting Properties

The proportion of amylose and amylopectin in the total starch content is closely related to the pasting properties of starch. Therefore, we determined the pasting properties of starch in rhizome of lotus at the later swelling stage under SCU and RCU treatments. Under SCU and RCU treatments, the starch paste performance of the lotus rhizome at the late swelling stage was significantly different in seven fertilization stages (Table 3). With the postponed application of SCU and RCU, peak viscosity, hot viscosity, breakdown viscosity and final viscosity were continuously increased and setback viscosity and peak temperature were persistently decreased. After SCU1 and RCU1 treatments, peak viscosity was significantly lower than other treatments, and setback viscosity was significantly higher than other treatments. These results suggested that the amylose content was relatively high under SCU1 and RCU1 treatments, which was consistent with the results of starch content measurement and starch particle count in the previous study.

### 2.6. Effects of SCU and RCU on Starch Synthesis Key Enzyme Activity

**Figure 5:**
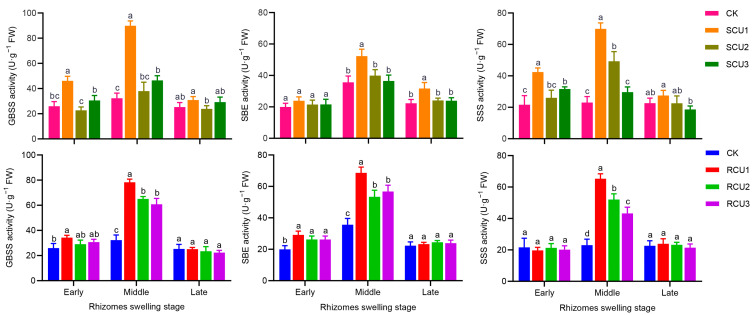
The activity of starch biosynthesis enzyme under SCU and RCU treatments. Different letters indicate a significant difference in starch biosynthesis enzyme activity at the swelling stage of the lotus rhizome between different treatments at the 5% level. Error bars show SD from three biological replicates.

In order to explore the effects of the SCU and RCU application stage on the mechanism of starch synthesis and accumulation, we determined the activities of key enzymes in starch synthesis. The application of SCU and RCU at different stages had a significant impact on GBSS, SBE and SSS activities. GBSS, SBE and SSS activity showed a single peak curve and increased in the middle swelling stage and then decreased in the late swelling stage (Figure 5). After SCU treatment, GBSS and SSS activity were significantly higher than other treatments, and they had no significant effect on SBE in the early swelling stage of the lotus rhizome. Under RCU treatment, GBSS and SBE activity were significantly higher than CK treatment, and they had no significant effect on SSS at the early swelling stage of the lotus rhizome. The highest peak activity of GBSS, SBE and SSS occurred in the middle swelling stage of SCU1 treatment, which was 178.08%, 46.72% and 203.06% higher, respectively, than CK treatment. After RCU1 treatment, GBSS, SBE and SSS reached peak values (78.26, 68.66 and 65.28 U·g^−1^, respectively), 142.07%, 92.54% and 183.33% higher, respectively, than CK treatment. SCU and RCU treatments had no significant effect on GBSS, SBE and SSS activity at the late swelling stage of the lotus rhizome. These results indicate that the application of SCU and RCU in the erect leaf stage can significantly increase the activities of GBSS, SBE and SSS.

### 2.7. Effects of SCU and RCU on Relative Expression of Key Enzyme Genes in Starch Synthesis

**Figure 6:**
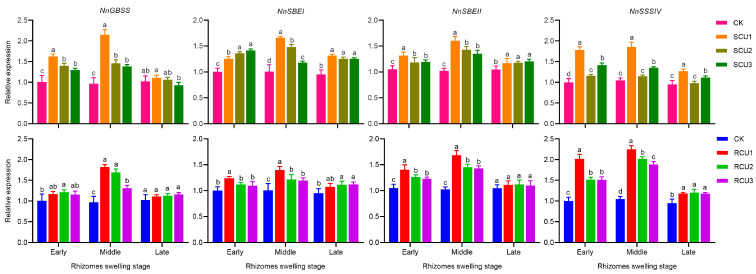
The relative expression level of starch biosynthesis enzyme genes under SCU and RCU treatments. Different letters indicate a significant difference in relative expression levels of starch biosynthesis enzyme genes at the swelling stage of the lotus rhizome between different treatments at the 5% level. Error bars show SD from three biological replicates.

To explain the effect of the SCU and RCU application stage on the regulatory mechanism of key enzyme activities in starch synthesis, we measured the relative expression levels of their corresponding genes. SCU and RCU treatments had a significant effect on key enzyme genes (*NnGBSS*, *NnSBEI*, *NnSBEII* and *NnSSSIV*) in starch synthesis (Figure 6). In the early stages of lotus rhizome swelling, the relative expressions of *NnGBSS*, *NnSBEII* and *NnSSSIV* under SCU1 treatment were significantly higher than under other treatments. In addition, the relative expressions of *NnSBEI*, *NnSBEII* and *NnSSSIV* under RCU1 treatment were significantly higher than under other treatments. SCU1 and RCU1 respectively increased the expression levels of *NnGBSS* by 121.65% and 87.93%, *NnSBEI* by 64.36% and 39.92%, *NnSBEII* by 56.86% and 65.29% and *NnSSSIV* by 77.88% and 116.35% in the middle swelling stage of lotus rhizome compared to CK. The relative expression of *NnSSSIV* under SCU1 treatment was significantly higher than other treatments in the late swelling stage of lotus rhizome. The relative expression of *NnGBSS*, *NnSBEI* and *NnSBEII* under SCU1 treatment was significantly higher than under CK treatment. Further, the expression level of *NnSSSIV* at the late swelling stage of the lotus rhizome was also higher with RCU1, RCU2 and RCU3 treatments than with CK treatment. These results indicated that SCU and RCU could significantly increase the relative expression levels of key enzyme genes in starch synthesis and promote the increase of key enzyme activities in starch synthesis.

### 2.8. Correlation Analysis of Key Enzyme Activities for Starch Synthesis and Starch Accumulation

As illustrated in Table 4, under SCU treatment, total starch content at the early swelling stage of lotus rhizome was significantly positively correlated with SBE activity and SSS activity. There were significant positive correlations between total starch content and GBSS activity and between amylopectin content and SSS activity, which were extremely significant positive correlations between amylose content and GBSS activity in the middle swelling stage of the lotus rhizome. SBE activity was significantly positively correlated with total starch content and amylose content of the lotus rhizome at the late swelling stage. After RCU treatment, total starch, amylose and amylopectin contents were negatively correlated with SSS activity in the early swelling stage of the lotus rhizome. The starch content of the lotus rhizome in the middle swelling stage was significantly positively correlated with the activities of key enzymes in starch synthesis. Total starch and amylopectin content were positively correlated with SSS activity at the late swelling stage of the lotus rhizome. These results indicate that GBSS, SBE and SSS are involved in starch synthesis and accumulation during rhizome swelling of lotus under SCU and RCU, but their action metabolic pathways are different.

## 3. Discussion

Previous studies have shown that GBSS, SBE and SSS are key enzymes in starch synthesis [39] and that changes in the activities of these enzymes could be divided into unimodality and bimodality after nitrogen fertilizer application in different stages [23,40]. Nitrogen fertilizer can alter transcriptional expression of key enzyme genes in crops and key enzyme activities for starch synthesis to increase starch content [41,42]. In this study, key enzyme activity (GBSS, SBE and SSS) and relative expression of key enzyme genes (*NnGBSS*, *NnSBEI*, *NnSBEII* and *NnSSSIV*) under SCU and RCU treatments all changed according to unimodal curves, which was consistent with rice results [43,44]. In previous studies, nitrogen fertilizer was found to alter starch synthesis enzyme activities and significantly increase relative expression of key enzyme genes at different stages in rice and wheat [45,46]. However, there are few reports on this aspect of slow-release fertilizer. Our results showed that the activity of three key enzymes and the relative expression of four key enzyme genes after SCU1 and RCU1 had their maximum value in the middle swelling stage of the lotus rhizome. Furthermore, the activity of GBSS and the relative expression of relevant isoforms (genes) *NnGBSS* were higher under SCU1 than under RCU1. After RCU1 treatment, SBE activity and relative expression of relevant isoforms (genes) *NnSBEII* were significantly higher than after SCU1 treatment. However, application at SCU and RCU (SCU2, SCU3, RCU2 and RCU3) significantly increased enzyme activity and gene expression of key enzymes in starch synthesis, but the effect was not as good as that of a one-time application of SCU and RCU in the erect leaf stage (SCU1 and RCU1), while delayed application of fertilizer would only increase labor costs. The results of this paper show that SCU1 and RCU1 significantly increased the activity of the key enzyme and gene expression of the key enzyme in starch synthesis, which promoted starch accumulation and increased starch content. SCU1 treatment promoted amylose accumulation and significantly increased amylose content in lotus rhizome.

Crop yield comes from biomass accumulation, and starch accumulation is one of the main methods of biomass accumulation, which is closely related to photosynthetic capacity [47,48]. Numerous studies have shown that the application of nitrogen fertilizer or slow-release fertilizer can significantly enhance the photosynthetic capacity of crops, promote starch accumulation and improve crop yield. In the study of proso millet, under the condition of nitrogen deficiency, the photosynthetic capacity and chlorophyll content of millet leaves were reduced, resulting in significantly reduced grain yield [49]. In addition, compared with other treatments, the net photosynthetic rate and chlorophyll content of sorghum leaves were significantly increased by applying nitrogen fertilizer at the jointing stage, and the yield was improved [50]. In buckwheat, the photosynthetic capacity, starch synthesis and grain yield of Tartary buckwheat were improved by nitrogen fertilizer treatment [51]. The application of slow-release urea can significantly increase the chlorophyll content and root area of rice, promote grain filling, and then increase rice yield [52,53]. In this study, SCU and RCU continuously promoted the increase of the SPAD value and Pn, which is conducive to increasing the starch content and thus increasing yield, and RCU1 treatment had the best effect on the increase of yield. SCU1 treatment had the best effect on increasing amylose content, which may be closely related to the extremely significant positive correlation between amylose content and GBSS activity in the lotus rhizome at the middle swelling stage under SCU treatment (Table 4). The results showed that the photosynthetic capacity of lotus leaves under RCU treatment remained at a high level for a longer time, e.g., 50–70 days, and more storage carbohydrates except starch were synthesized, thus increasing yield, which may be closely related to the N release rate of RCU showing a sustained release during the 10–70 days (Figure 1). Furthermore, delayed application of SCU and RCU could reduce the photosynthetic capacity and starch content of lotus leaves and significantly reduce lotus rhizome yield.

Starch pasting properties reflect the degree of expansion of starch granules [54]. At high temperatures, amylopectin is more likely to expand and break down than amylose, while amylose rearrangement occurs in the cooling process [55,56]. Therefore, high viscosity means more amylopectin content, while high setback viscosity means more amylose content. In sorghum research, the application of nitrogen fertilizer at the jointing stage can significantly improve the peak viscosity and final viscosity of starch [57]. Furthermore, compared with treatments using high-level nitrogen fertilizers, moderate nitrogen fertilizers significantly increased peak viscosity and final viscosity of starch in common buckwheat [58]. In waxy maize, the peak viscosity, final viscosity and setback viscosity of starch in grain increased gradually with the delayed application of slow-release fertilizer, where a one-time application of slow-release fertilizer at six leaf stages significantly increased peak viscosity, final viscosity and setback viscosity of grain starch, which made the grain taste waxier and softer [28]. In our study, delayed application of SCU and RCU significantly increased peak viscosity, final viscosity and setback viscosity of lotus rhizome starch, which were significantly reduced under SCU1 and RCU1 treatments (Table 3). After SCU1 treatment, the main reason that the rhizome starch peak viscosity was significantly lower than after RCU1 treatment at the late swelling stage was that amylose content under SCU1 treatment was significantly higher than under RCU1 treatment, while amylopectin content was significantly lower under RCU1 treatment (Table 2), which was consistent with the study results in waxy maize. The shape and number of starch granules are also the main indexes affecting the physicochemical properties of starch. In the study of super rice, under moderate nitrogen application, starch granule size was significantly increased compared to other treatments [59]. After nitrogen fertilizer treatment, the number of starch grains in the endosperm of Tartary buckwheat increased significantly, but the grain size of starch did not change significantly [60]. In this study, delayed application of SCU and RCU had no significant effect on starch granule morphology, but the number of starch granules gradually decreased (Figure 4). This phenomenon may be caused by the delayed application of SCU and RCU, which were not conducive to improving the key enzyme activities of starch synthesis and expression of related genes during the rhizome swelling stage (Figure 5 and Figure 6), and which decreased the accumulation of amylose and amylopectin (Figure 3). In addition, the contents of amylose and amylopectin were significantly reduced at the later stage of rhizome enlargement and the number of starch granules was eventually reduced. This is consistent with previous studies on Tartary buckwheat. However, SCU1 and RCU1 treatments significantly increased the number of long oval starch granules and nearly round starch granules. These results indicate that SCU1 and RCU1 can promote starch synthesis and accumulation, significantly increase the number of starch particles, reduce starch pasting properties and make lotus rhizome crunchier and tastier in the cooking process by regulating key starch synthesis enzymes and the expression of related genes.

## 4. Materials and Methods

### 4.1. Experimental Materials

The field experiment was carried out at the aquatic vegetable test field of Yangzhou University in Yangzhou City, Jiangsu Province, in 2022. The lotus cultivars used in the experiments were ‘MRH’. Two SRFs were selected for the experiment, including SCU: sulfur-coated compound fertilizer (the main slow-release component is sulfur-coated urea) (N/P_2_O_5_/K_2_O = 20%/10%/18%) and RCU: resin-coated urea (N ≥ 43%). SCU and RCU release periods were 60 and 90 days.

### 4.2. Experimental Design

The experiment was conducted in a pool with an area of 11.02 m^2^ (5.8 m × 1.9 m). The nutrient content of the soil prior to sowing was as follows: 12.8 g·kg^−1^ organic matter, 1.04 g·kg^−1^ available nitrogen, 9.4 mg·kg^−1^ available phosphorus, 54.4 mg·kg^−1^ available potassium. The available nitrogen, available phosphorus, available potassium and organic matter in soils were measured with alkaline hydrolysis diffusion method, molybdenum-antimony colorimetry method, flame photometric of ammonium acetate method and potassium dichromate oxidation method, respectively [61]. Lotus was planted on 4 May 2022. The optimal total nitrogen (total N = 207 kg·ha^−1^) was selected by referring to our previous method [62]. Seven fertilization modes were designed. (1) CK (zero nitrogen fertilizer application) and slow-release fertilizer, (2) SCU1, (3) SCU2 and (4) SCU3: 1035 kg·ha^−1^ (total N = 207 kg·ha^−1^) sulfur-coated compound fertilizer was applied once at May 25 (erect leaf stage), June 20 (erect leaf completely covering the water stage) and July 10 (swelling stage of lotus rhizomes), respectively. (5) RCU1, (6) RCU2 and (7) RCU3: 482 kg·ha^−1^ (total N = 207 kg·ha^−1^) resin-coated urea was applied once at May 25 (erect leaf stage), June 20 (erect leaf completely covering the water stage) and July 10 (swelling stage of lotus rhizomes), respectively. The phosphorus and potassium levels of the seven treatments were the same: total P = 103.5 kg·ha^−1^ and total K = 186.3 kg·ha^−1^, respectively. The difference part was supplemented by superphosphate (P_2_O_5_ ≥ 16%) and potassium sulfate (K_2_O ≥ 52%).

### 4.3. N Release Characteristics of SCU and RCU

N release from SCU and RCU was studied in a pool under the same conditions as the lotus planting. Nitrogen release after fertilization was studied by soil embedding [63]. The fertilizer weighing 10 g was placed into a nylon gauze bag (120 mm × 60 mm) with a hole diameter of 0.15 mm. The bags were buried 10 cm below the surface of the soil. Three bags were sampled at 10, 20, 30, 40, 50, 60, 70, 80 and 90 days after application. The collected samples were rinsed with distilled water and were put into a 30 °C oven for drying until reaching a constant dry weight, weighed and then the nitrogen release was calculated.

### 4.4. SPAD Value and Pn

Five identical erect leaves were selected for each treatment, and three points were randomly selected for each erect leaf, and their growth status was measured at 0, 10, 20, 30, 40, 50 and 60 days after SCU application and 0, 10, 20, 30, 40, 50, 60, 70, 80 and 90 days after RCU application to measure the leaf net photosynthetic rate using the LI-6400 xt photosynthetic apparatus (LI-COR company, Lincoln, NE, USA). Leaf SPAD values were measured using a chlorophyll meter (SPAD-502, Soil-Plant Analysis Development, Konika Minolta, Tokyo, Japan).

### 4.5. Plant Sample Harvesting

Lotus rhizomes were collected in three stages: early (the rhizomes have two segments and the one containing the terminal bud is greater than 3 cm in diameter), middle (the rhizomes have three segments and the one containing the terminal bud is greater than 3 cm in diameter) and late (the rhizomes have four or more segments and the segment containing the terminal bud is greater than 3 cm in diameter) swelling stage, and sample harvesting times were 10 August, 30 August and 25 September, respectively in 2022 [4]. A portion of each sample was washed, rapidly frozen in liquid nitrogen and stored at −80 °C to RNA extraction and enzyme activity related to starch synthesis was determined. Another portion of each sample was fixed at 105 °C for 30 min, dried at 80 °C to constant weight and then used to determine starch and amylose content. Lotus rhizomes were harvested and the yield measured on 15 October 2022.

### 4.6. Starch Content

Extraction of total starch and amylose content refers to the anthrone-sulfuric acid colorimetry method with slight modification [62]. The lotus rhizome samples stored in the refrigerator at −80 °C were ground thoroughly, and the powder obtained from each treatment was weighed into three parts, each part 0.03 g, to determine the total starch content of the lotus rhizome. The total starch content was determined by anthrone colorimetry using a starch content kit (Beijing Solarbio Science & Technology Co., Ltd., No. BC0700 Beijing, China). Lotus rhizome samples were fixed at 105 °C for 30 min, dried at 80 °C to constant weight, thoroughly ground the lotus rhizome sample and three servings weighed for each treatment, each part 0.01 g, and then used to determine amylose content. The content of amylose was determined by iodine colorimetry using an amylose content kit (Beijing Solarbio Science & Technology Co., Ltd., No. BC4260 Beijing, China). Amylopectin content = total starch content − amylose content.

### 4.7. Starch Isolation

The starch was isolated with reference to Zhou et al., 2020 with minor modifications [59]. Lotus rhizomes were soaked in NaOH (pH 7.5) solution for 4 h and then homogenized with a tissue homogenizer for 1 min. The homogenate was passed through a 200-mesh sieve, the filtrated mixture was collected in a 50 mL centrifuge tube and then 100 mg alkaline protease and 20 μL preservative was added for digestion at 42 °C for 6 h in a table concentrator. After digestion, the samples were centrifuged at 3000 rpm for 10 min.

### 4.8. Starch Granule Morphology

The starch was placed in the alcohol and dispersed evenly with an ultrasonic oscillator. Next, a little alcohol was glued to a toothpick to leave a uniform layer of powder on the surface of the sample table and the powder was dried with hot air electricity. The morphology of starch particles was observed by environmental scanning electron microscope (XL-30ESEM, Philips, Amsterdam, Holland) and we examined the number of starch particles by using statistics.

### 4.9. Starch Pasting Properties

Starch pasting properties were determined using a rapid viscosity analyzer (Model 3D, Newport Scientific, Warriewood, NSW, Australia). Starch samples were 2.5 g total weight; 7%, *w*/*w*, dry basis and mixed with 25 g H_2_O. The programming cycle for pasting was set at 14 min. Starch samples were started at 50 °C for 1 min and then heated from 50 °C to 95 °C, held at 95 °C for 2.5 min, cooled to 50 °C at 12 min and finally held for 2 min [4].

### 4.10. Key Enzyme Activity in Starch Synthesis

The activities of GBSS, SBE and SSS were measured by starch synthase kits (Beijing Solarbio Chemical Co., Ltd., Beijing, China, No. BC3290, BC1860 and BC1850). About 0.1 g lotus rhizome sample was weighed into the mortar, then 1 mL extract added, homogenize fully in the ice bath; 10,000× *g* centrifuged at 4 °C for 10 min, supernatant was discarded, 1 mL of extract was added to precipitation and thoroughly mixed and then placed on ice for use in the test [4].

### 4.11. Relative Expression of Key Enzyme Genes in Starch Synthesis

Lotus samples were ground into fine powder in liquid nitrogen, loaded into a centrifugal tube and stored in a −80 °C refrigerator. Three biological replicates were used for RNA extraction per treatment. RNA was obtained using the RNA extraction kit (PD Biotech, Shanghai, China). Then, HiScriptII RT SuperMixfor qPCR (Vazyme Biotech Co., Ltd. Nanjing, China) was used for reverse transcription into cDNA. The RT-qPCR was conducted on a CFX96 real-time PCR system (Bio-Rad, Hercules, CA, USA) on a total reaction volume of 20 μL (consisting of 10 μL Hieff UNICON^®^ Universal Blue qPCR SYBR Green Master Mix (Cat No.11184; YEASEN, Shanghai, China), 0.4 μL forward primer, 0.4 μL reverse primer, 1 μL diluted cDNA and 8.2 μL RNase-Free ddH_2_O). The RT-qPCR data were analyzed using the 2^−ΔΔCT^ method [64].

### 4.12. Statistical Analysis

Microsoft Excel 2016 software was used to organize the data. Graphpad Prism 8.0 was used to construct the graphs. Data were analyzed statistically using IBM SPSS Statistics 26.0 software, *p* values of <0.05 were considered statistically significant (*) and *p* values of <0.01 were considered highly statistically significant (**). All data were expressed as mean ±SD.

## 5. Conclusions

A one-time application at the erect leaf stage of both SCU and RCU is suitable for high quality and high yield of lotus rhizome production. These results provide a technical choice for the one-time application of slow-release fertilizer in lotus rhizome production and cultivation.

## Figures and Tables

**Table 1:** The SPAD value and Pn under SCU and RCU treatment.

SPAD/Pn	Treatment	Day after SCU/RCU Treatment									
		0d	10d	20d	30d	40d	50d	60d	70d	80d	90d
SPAD	CK	34.20 ± 1.40 ^cd^	34.60 ± 1.14 ^d^	35.25 ± 1.07 ^e^	36.60 ± 0.94 ^e^	36.15 ± 1.46 ^e^	34.60 ± 0.97 ^f^	34.15 ± 1.32 ^g^	35.25 ± 1.14 ^d^	34.65 ± 0.99 ^d^	34.75 ± 0.97 ^d^
	SCU1	34.95 ± 0.83 ^ab^	35.65 ± 0.49 ^c^	40.35 ± 0.49 ^a^	45.95 ± 0.83 ^a^	47.00 ± 1.03 ^a^	44.75 ± 0.97 ^b^	41.15 ± 0.88 ^e^	-	-	-
	SCU2	34.65 ± 0.49 ^bc^	36.40 ± 0.94 ^a^	38.95 ± 0.89 ^b^	40.95 ± 0.83 ^b^	44.05 ± 0.83 ^b^	43.70 ± 0.47 ^c^	41.95 ± 0.94 ^d^	-	-	-
	SCU3	33.60 ± 1.27 ^d^	34.65 ± 0.49 ^d^	37.25 ± 0.72 ^d^	38.40 ± 0.50 ^d^	42.40 ± 0.60 ^c^	38.95 ± 0.89 ^e^	36.30 ± 0.66 ^f^	-	-	-
	RCU1	34.90 ± 0.85 ^b^	35.80 ± 0.77 ^bc^	37.60 ± 1.05 ^cd^	40.00 ± 0.65 ^c^	42.60 ± 0.88 ^c^	45.20 ± 0.89 ^ab^	46.00 ± 1.45 ^b^	48.40 ± 1.39 ^a^	44.80 ± 0.77 ^a^	42.20 ± 0.77 ^a^
	RUC2	35.60 ± 0.82 ^a^	36.60 ± 0.50 ^a^	38.00 ± 0.92 ^c^	41.05 ± 0.89 ^b^	43.00 ± 0.92 ^c^	45.65 ± 0.88 ^a^	46.80 ± 1.20 ^a^	43.80 ± 1.20 ^b^	41.70 ± 0.98 ^b^	38.80 ± 1.20 ^b^
	RCU3	35.60 ± 1.05 ^a^	36.20 ± 1.20 ^ab^	37.20 ± 0.77 ^d^	38.20 ± 0.77 ^d^	39.40 ± 1.05 ^d^	42.05 ± 0.83 ^d^	44.50 ± 1.00 ^c^	42.70 ± 1.22 ^c^	38.70 ± 1.56 ^c^	36.60 ± 1.05 ^c^

Pn(μmol m^−2^ s^−1^)	CK	1.78 ± 0.10 ^bcd^	1.91 ± 0.09 ^b^	1.88 ± 0.08 ^d^	1.91 ± 0.09 ^f^	1.94 ± 0.10 ^e^	1.93 ± 0.05 ^d^	1.89 ± 0.09 ^e^	1.95 ± 0.10 ^d^	1.98 ± 0.10 ^c^	1.95 ± 0.11 ^c^
	SCU1	1.86 ± 0.05 ^a^	2.06 ± 0.05 ^a^	2.28 ± 0.08 ^a^	2.46 ± 0.05 ^a^	2.74 ± 0.05 ^a^	2.43 ± 0.05 ^a^	2.16 ± 0.05 ^c^	-	-	-
	SCU2	1.81 ± 0.07 ^abc^	2.00 ± 0.07 ^a^	2.08 ± 0.08 ^b^	2.26 ± 0.05 ^b^	2.47 ± 0.08 ^b^	2.34 ± 0.05 ^b^	2.02 ± 0.06 ^d^	-	-	-
	SCU3	1.84 ± 0.12 ^ab^	1.89 ± 0.07 ^b^	1.97 ± 0.05 ^c^	2.04 ± 0.05 ^d^	2.35 ± 0.05 ^c^	2.23 ± 0.08 ^c^	1.93 ± 0.07 ^e^	-	-	-
	RCU1	1.78 ± 0.08 ^bcd^	1.92 ± 0.09 ^b^	2.02 ± 0.06 ^bc^	2.12 ± 0.06 ^c^	2.30 ± 0.09 ^c^	2.30 ± 0.07 ^b^	2.43 ± 0.07 ^a^	2.62 ± 0.08 ^a^	2.45 ± 0.05 ^a^	2.39 ± 0.09 ^a^
	RCU2	1.75 ± 0.05 ^cd^	1.87 ± 0.09 ^b^	1.96 ± 0.05 ^c^	2.00 ± 0.07 ^de^	2.06 ± 0.10 ^d^	2.22 ± 0.08 ^c^	2.27 ± 0.08 ^b^	2.33 ± 0.07 ^b^	2.21 ± 0.09 ^b^	2.16 ± 0.07 ^b^
	RCU3	1.73 ± 0.05 ^d^	1.89 ± 0.09 ^b^	1.88 ± 0.09 ^d^	1.97 ± 0.08 ^e^	1.97 ± 0.09 ^e^	1.95 ± 0.10 ^d^	2.12 ± 0.08 ^c^	2.23 ± 0.08 c	2.19 ± 0.07 ^b^	2.10 ± 0.08 ^b^

Values in the same column with different letters are significantly different (*p* < 0.05). Data are presented as the mean ± SD from fifteen biological replicates.

**Table 2:** Starch content and yield under SCU and RCU treatments.

Treatment	Total Starch Content/% FW			Amylose Content/% FW			Amylopectin Content/% FW			Yield/kg
	Early	Middle	Late	Early	Middle	Late	Early	Middle	Late	
CK	5.80 ± 0.16 ^d^	8.60 ± 0.22 ^c^	15.54 ± 0.25 ^cd^	2.15 ± 0.08 ^c^	3.30 ± 0.10 ^cd^	4.81 ± 0.15 ^c^	3.65 ± 0.10 ^a^	5.30 ± 0.11 ^cd^	10.73 ± 0.10 ^bcd^	27.92 ± 3.93 ^e^
SCU1	6.58 ± 0.08 ^a^	9.38 ± 0.18 ^ab^	16.58 ± 0.17 ^a^	2.74 ± 09 ^a^	3.85 ± 0.87 ^a^	5.68 ± 0.19 ^a^	3.84 ± 0.05 ^a^	5.54 ± 0.26 ^bcd^	10.90 ± 0.31 ^bc^	44.35 ± 1.55 ^b^
SCU2	6.02 ± 0.13 ^cd^	8.61 ± 0.16 ^c^	15.29 ± 0.15 ^d^	2.39 ± 0.15 ^b^	3.23 ± 0.11 ^d^	4.94 ± 0.14 ^c^	3.63 ± 0.28 ^a^	5.38 ± 0.06 ^cd^	10.36 ± 0.29 ^d^	35.63 ± 1.44 ^c^
SCU3	6.11 ± 0.16 ^bc^	8.60 ± 0.17 ^c^	15.36 ± 0.10 ^d^	2.25 ± 0.13 ^bc^	3.33 ± 0.06 ^cd^	4.73 ± 0.05 ^c^	3.86 ± 0.24 ^a^	5.27 ± 0.22 ^d^	10.64 ± 0.14 ^cd^	37.92 ± 2.03 ^c^
RCU1	6.34 ± 0.15 ^ab^	9.67 ± 0.21 ^a^	16.75 ± 0.17 ^a^	2.35 ± 0.07 ^b^	3.52 ± 0.08 ^b^	5.22 ± 0.10 ^b^	3.99 ± 0.22 ^a^	6.15 ± 0.17 ^a^	11.53 ± 0.20 ^a^	47.06 ± 3.56 ^a^
RCU2	6.15 ± 0.17 ^bc^	9.32 ± 0.16 ^b^	16.01 ± 0.13 ^b^	2.40 ± 0.05 ^b^	3.46 ± 0.11 ^bc^	4.92 ± 0.13 ^c^	3.75 ± 0.18 ^a^	5.86 ± 0.24 ^a^	11.09 ± 0.25 ^b^	37.53 ± 3.52 ^c^
RCU3	6.09 ± 0.21 ^bc^	9.10 ± 0.20 ^b^	15.71 ± 0.17 ^c^	2.27 ± 0.06 ^bc^	3.46 ± 0.09 ^bc^	4.88 ± 0.16 ^c^	3.82 ± 0.20 ^a^	5.64 ± 0.11 ^bc^	10.83 ± 0.04 ^bc^	32.02 ± 2.24 ^d^

Values in the same column with different letters are significantly different (*p* < 0.05). Data are presented as the mean ± SD from three biological replicates.

**Table 3:** Effects of SCU and RCU on starch pasting properties in the rhizomes of the lotus.

Treatment	PV (cP)	HV (cP)	BV (cP)	FV (cP)	SV (cP)	P_time_ (min)	P_temp_ (°C)
CK	6781 ± 28 ^c^	2112 ± 29 ^c^	4669 ± 11 ^de^	2572 ± 33 ^cd^	460 ± 7 ^c^	3.52 ± 0.02 ^cd^	73.83 ± 0.03 ^c^
SCU1	6545 ± 25 ^e^	1856 ± 11 ^e^	4689 ± 61 ^cd^	2484 ± 13 ^e^	628 ± 33 ^a^	3.64 ± 0.07 ^a^	74.65 ± 0.27 ^a^
SCU2	6886 ± 12 ^b^	2164 ± 30 ^c^	4722 ± 28 ^bc^	2718 ± 33 ^b^	554 ± 25 ^b^	3.53 ± 0.03 ^c^	74.15 ± 0.10 ^b^
SCU3	7043 ± 30 ^a^	2321 ± 11 ^a^	4822 ± 38 ^a^	2794 ± 49 ^a^	473 ± 11 ^c^	3.47 ± 0.04 ^d^	73.15 ± 0.07 ^e^
RCU1	6630 ± 55 ^d^	1986 ± 59 ^d^	4644 ± 56 ^e^	2536 ± 46 ^de^	550 ± 13 ^b^	3.59 ± 0.05 ^b^	74.17 ± 0.11 ^b^
RCU2	6982 ± 37 ^a^	2246 ± 34 ^b^	4736 ± 35 ^b^	2603 ± 33 ^c^	357 ± 17 ^d^	3.47 ± 0.03 ^d^	73.53 ± 0.13 ^d^
RCU3	6840 ± 50 ^bc^	2164 ± 36 ^c^	4676 ± 14 ^de^	2613 ± 26 ^c^	449 ± 12 ^c^	3.54 ± 0.02 ^c^	73.65 ± 0.13 ^cd^

Values in the same column with different lowercase letters are significantly different (*p* < 0.05). PV, peak viscosity; HV, hot viscosity; BV, breakdown viscosity (PV–HV); FV, final viscosity; SV, setback viscosity (FV–HV); P_time_, peak time; P_temp_, pasting temperature. Data are presented as the mean ± SD from three biological replicates.

**Table 4:** Correlation analysis of key enzyme activities for starch synthesis and starch accumulation.

Starch Content	Key Enzymes	SCU Treatment			RCU Treatment		
		Early	Middle	Late	Early	Middle	Late
Total starch content	GBSS	0.92	0.97 *	0.71	0.94	0.89 *	0.32
	SBE	0.99 **	0.98 **	0.93 *	0.99 **	0.85 *	0.21
	SSS	0.96 *	0.86	0.87	−0.78	0.98 **	0.78 *
Amylose content	GBSS	0.82	0.98 **	0.58	0.62	0.88 *	0.32
	SBE	0.86	0.83	0.67 *	0.84	0.78 *	0.71 *
	SSS	0.88	0.77	0.62	−0.37	0.76 *	0.13
Amylopectin content	GBSS	0.74	0.87	0.77	0.99 **	0.83 *	0.31
	SBE	0.64	0.98 **	0.60	0.90	0.87 *	0.25
	SSS	0.80	0.96 *	0.52	−0.94	0.92 **	0.81 *

(*) represents *p* values of < 0.05 and is considered statistically significant. (**) represents *p* values of < 0.01 and is considered highly statistically significant.

## Data Availability

Not applicable.
